# From Stressful Experiences to Depression in Chinese Migrant Children: The Roles of Stress Mindset and Coping

**DOI:** 10.3389/fpsyg.2021.601732

**Published:** 2021-04-06

**Authors:** Luxi Chen, Li Qu

**Affiliations:** ^1^Centre for Family and Population Research, National University of Singapore, Singapore, Singapore; ^2^School of Social Sciences, Nanyang Technological University, Singapore, Singapore

**Keywords:** migrant children, stress mindset, threat, challenge, approach coping, avoidant coping, depression, gender

## Abstract

Migrant children are at high risk for depression, though the exact mechanism is still unclear. This study investigated whether and how different stress mindsets (threat vs. challenge) and coping strategies (avoidant vs. approach) mediated the association between stressful experiences and depression in migrant children, and whether these relationships would be moderated by gender. One hundred and ninety-eight rural-to-urban migrant children (56.0% girls; *M_age_* = 11.8 years) in Beijing, China, completed self-administered measures of stressful experiences, threat and challenge mindsets, coping strategies, and depression. Path analysis was conducted to examine the proposed mediation model. A dual-pathway model of stress coping was discovered: (1) a stress-threat-avoidance-depression pathway, in which threat mindset and avoidant coping mediated the association between stressful experiences and depression, and (2) a challenge-approach-enhancement pathway, in which approach coping mediated the association between challenge mindset and fewer depressive symptoms, without being influenced by stressful experiences. The dual-pathway mechanism did not vary by gender, and it can explain the greater vulnerability of girls to depression. Together, findings suggest that stressful events, threat mindset, and avoidant coping act as risk factors for depression, whereas challenge mindset and approach coping can function as protective factors to counteract the impacts of stressful experiences and promote psychological well-being among migrant children.

## Introduction

Migration, a change in the location of residence for better living conditions, has been a universal phenomenon (Bhugra, 2004). With globalization and economic development, the number of migrant children who are relocated to big cities with their parents has been growing dramatically (Crosnoe and Fuligni, 2012; Hu et al., 2014). For example, in China, there were 34.26 million migrant children and adolescents aged from 0 to 17 in 2015, accounting for approximately 12.8% of the children population in the nation, estimated by the National Working Committee on Children Women under the State Council (NWCCW), National Bureau of Statistics of the People’s Republic of China (NBS), United Nations Children’s Fund (UNICEF) (2018). In particular, the number of primary school-aged migrant children in Beijing was estimated to be approximately 366,000, comprising about 44.6% of all the primary school students in this city, according to the Beijing Municipal Bureau of Statistics and NBS Survey Office in Beijing (2015). Globally, migration has been associated with more negative life events and elevated risks for emotional and adjustment problems in children and adolescents (Stevens and Vollebergh, 2008; Jordan and Graham, 2012). Compared with typically developing children in China, rural-to-urban migrant children experienced more stressful life events in education, health care, and adaption (Wen and Lin, 2012), and they were at higher risk of depression (Hu et al., 2014; Wang and Mesman, 2015). Although theoretically, one’s interpretation of stress and the selection of coping strategies mediate the association between the experiences of stressful events and depression (Lazarus and Folkman, 1984, 1987; Lazarus, 1991, 2006), it is unclear whether it is the same case for rural-to-urban migrant children in China. Therefore, it is necessary to investigate whether the association between stressful experiences and depression among migrant children would be mediated by stress interpretation and stress coping, and if any, what factors may moderate the mechanism.

The impacts of negative life events on Chinese migrant children’s depression has been well-established in previous work (Wang and Mesman, 2015; Ye et al., 2016; Jiang et al., 2019). Migration is a stressful process. Stress occurs when the demands of the external environment exceed an individual’s inner competence (Lazarus and Folkman, 1984). In China, migrant workers in urban areas generally live in insecure conditions, and receive lower income and fewer welfare benefits compared with nonmigrant residents (Wong et al., 2007). Children who migrated with their parents from rural areas to cities usually suffer from poverty, and hardly have access to public services for primary education and health care (Wen and Lin, 2012). For instance, under the management of the Household Registration System (i.e., *hukou* system) in China, many of the migrant children in Beijing are enrolled in private migrant schools, which are usually located in the outskirts of the city with a limited number of qualified teachers and educational resources (Chen and Feng, 2013). In general, rural-to-urban migrant children are exposed to more stressful events such as economic stress, academic challenges, family conflicts, peer victimization, and adaption problems compared with ordinary children (Wang and Mesman, 2015; Ye et al., 2016). As a result, migrant children were more vulnerable to mental health problems such as depression and loneliness than their local counterparts in urban areas (Chen et al., 2014; Hu et al., 2014). The prevalence of depression in this special population was much higher than that in the average Chinese children in China (Zhou et al., 2018; Rao et al., 2019).

According to the transactional theory of stress coping (Lazarus and Folkman, 1984, 1987) and the appraisal theory of emotion (Lazarus, 1991, 2006), the relationship between stressful experiences and emotions is accounted for by how an individual interprets stress and copes with stress. Moreover, the model of resilience (Garmezy et al., 1984; Masten et al., 1990) highlighted the important roles of individual-level protective factors such as positive appraisal and adaptive coping in counteracting the negative impacts of adversities and promoting psychological well-being. In general populations, the negative impacts of stressful events on psychological well-being can be mitigated by resilience, which embodies positive interpretational style and adaptive coping (Fergus and Zimmerman, 2005; Bonanno and Diminich, 2013; Zimmerman et al., 2013), and a positive stress mindset that acknowledges the opportunities for personal growth inherent in stress (Huebschmann and Sheets, 2020). Specifically among Chinese migrant children, the negative impact of stressful events on depression was less observed in children with greater resilience (Ye et al., 2016) and a stress-is-enhancing mindset (Jiang et al., 2019). Together, this line of research suggests that different stress mindsets and coping styles play essential roles in mediating or mitigating the relationship between stressful experiences and psychological well-being.

Stress mindset is a meta-cognitive belief about the enhancing or debilitating nature of stressful events in general (Crum et al., 2013, 2017). According to the biopsychosocial (BPS) model of challenge and threat (Blascovich and Tomaka, 1996; Blascovich et al., 2000), people in a *threat* state tend to anticipate damage or harm from stressful events, while people in a *challenge* state tend to anticipate gains and growth from overcoming obstacles. In this study, we conceptualized the belief that stressful events are threatening and debilitating, in general, as a *stress-is-a-threat* mindset (hereafter, we called it *threat mindset*), and we conceptualized the belief that stress provides opportunities of personal growth as a *stress-is-a-challenge* mindset (hereafter, we called it *challenge mindset*). Interpreting stress as a threat has been associated with more emotional symptoms such as depression, anxiety, and distress, whilst interpreting stress as a challenge has been linked to positive psychological well-being (e.g., Lazarus, 2006; Seery, 2011). The different influences of threat and challenge appraisals on psychological well-being have been discovered in nonclinical samples (Tomaka et al., 1993; Blascovich and Tomaka, 1996), clinical samples (Ellis et al., 2009), as well as vulnerable children and adolescents (Bryant et al., 2007; Meiser-Stedman et al., 2009). Among rural-to-urban migrant children in China, negative automatic thoughts mediated the relationship between stressful experiences and depression (Liu et al., 2017); in contrast, a stress-is-enhancing mindset mitigated the impacts of negative life events on depressive symptoms (Jiang et al., 2019). Hence, it is plausible that threat mindset may mediate the association between stressful experiences and depression levels in migrant children, whereas challenge mindset may protect them from depression.

Coping, referring to one’s cognitive and behavioral efforts that aim at managing stress, is another important process that influences one’s emotional outcomes (Lazarus and Folkman, 1984). Broadly speaking, coping strategies can be categorized as two styles, namely, *approach coping* (such as taking direct actions to resolve problems, seeking support, and confronting emotional response to a stressor), and *avoidant coping* (such as denial, withdrawal, and self-blaming) (Suls and Fletcher, 1985; Roth and Cohen, 1986; Skinner et al., 2003). In adolescents and youth, using more avoidant coping strategies and fewer approach coping strategies predicted higher emotional distress and depression in both nonclinical samples (Herman-Stabl et al., 1995; Seiffge-Krenke and Klessinger, 2000) and clinical samples (Dempsey et al., 2000). The mediating role of coping in the association between negative life events and depression has also been found in vulnerable children who suffered from domestic violence, disasters, or major life events (Dempsey et al., 2000; Lengua and Long, 2002; Fortin et al., 2011). In rural-to-urban migrant children in China, greater resilience and the ability to cope with stressful situations effectively can reduce their risk for depression (Ye et al., 2016). Thus, it is reasonable to expect that approach and avoidant coping strategies may contribute to the emotional outcomes of this special population.

Emotional disorders such as anxiety and depression were products of the interaction between stress interpretational styles and coping patterns (Williams, 2002). A stress-is-enhancing mindset (that acknowledges the opportunities for personal growth inherent in stress) is associated with approach-motivated responses, whereas a stress-is-debilitating mindset (that focuses on the damage and threat posed by the stressful events) is associated with avoidance-motivated responses (see Jamieson et al., 2018, for a review). The association between threat appraisal and avoidant coping, and that between positive stress appraisal and active coping, has also been established in children and adolescence (Lengua et al., 1999; Lengua and Long, 2002; Zalewski et al., 2011). However, few studies have investigated the exact mechanism regarding how different stress mindsets (challenge vs. threat) and coping strategies (approach vs. avoidant) work with each other to account for the influence of stressful experiences on depression in migrant children in China.

Gender differences in depression and stress coping are well documented in the literature. Females are more vulnerable to depression (see Nolen-Hoeksema, 2001, for a review), with twofold higher rates of depression than males (Zahn-Waxler et al., 2005). According to the Cognitive Vulnerability-Transactional Stress Depression Model (Hankin and Abramson, 2001), the higher risk of females for depression can be explained by their greater exposure to negative life events, negative stress appraisal, and maladaptive coping strategies. Among typically developing children and adolescents, girls show more depressive symptoms than boys due to their greater exposure to stressful events, hormonal changes, sexual maturity, interpersonal changes, and expectations (e.g., Weiss et al., 1999; Wade et al., 2002; Matud, 2004; Hankin et al., 2008). Girls also tend to perceive stressful events as more threatening (Macnamara and Rupani, 2017; Park et al., 2018) and adopt maladaptive coping strategies (e.g., rumination that focuses on loss and disruptions rather than on solutions) to deal with stressful situations (Matud, 2004). However, the pattern among migrant children is less clear. For example, Jiang et al. (2019) found that girls showed more depressive symptoms than boys (*M_age_* = 11.8, SD = 1.16, range: 10–14 years), but girls and boys were equal in Liu et al.’s (2017) study with a younger sample (*M_age_* = 9.47, SD = 1.46, range: 7–14 years). It is possible that gender difference in depression may be more significant among young teens, but less significant among children and pre-teens. Indeed, in general samples, gender difference in depression emerges in early adolescence (Twenge and Nolen-Hoeksema, 2002; Wade et al., 2002; Merikangas et al., 2010), although gender difference in sadness can occur as early as in preschool period among vulnerable children (Luby et al., 2009). Relatively, little is known about whether gender differences in depression and stress coping process would differ between migrant children, preteens, and young teens.

Additionally, there have been mixed findings on the moderating role of gender in the association between stressful life events and depression levels. Gender moderated the influences of daily stress and certain coping strategies on depression in some studies (e.g., Seiffge-Krenke and Stemmler, 2002; Rodríguez-Naranjo and Caño, 2016), but not in others (e.g., Kort-Butler, 2009). Specifically in Chinese migrant children, Jiang et al. (2019) found that gender did not moderate the relationship between stressful experiences and depression, although it moderated the influence of stressful experiences on life satisfaction. It requires further examination to investigate whether the pathways to depression vary by gender in migrant children.

To fill the gap in understanding the mechanism by which stressful experiences influence depression in migrant children, the current study examined whether threat and challenge mindsets work with approach and avoidant coping strategies to mediate the association between stressful experiences and depression, and whether gender moderates any path in the mediation model. We derived the following hypotheses from the literature: (1) stressful experiences would be positively associated with threat mindset, avoidant coping, and depression levels, and negatively associated with challenge mindset and approach coping; (2) the association between stressful experiences and depression would be mediated by threat mindset and (reduced) challenge mindset; (3) avoidant coping and approach coping would mediate the relationship between stress mindsets and depression. Additionally, it was expected that (4) gender differences would be observed in all the main variables, and in particular, (4a) girls would report more stressful experiences, higher levels of threat mindset, lower levels of challenge mindset, more avoidant coping strategies, fewer approach strategies, and more depressive symptoms, and (4b) there would be an interaction effect of gender and age, with gender difference being significant for the older age group. Finally, we hypothesized that (5) gender would moderate the paths we described in (1), (2), and (3).

## Materials and Methods

### Participants

Participants were a convenient sample of 200 migrant children recruited from the 4th to 6th grades of two private migrant schools in the Tongzhou and Daxing Districts of Beijing, China. Migrant children who (1) migrated with their parents to Beijing and (2) had no household register (i.e., *hukou*) in Beijing were eligible for this study. Two children were excluded from the final data analysis for they had more than four missing responses in the measure of depression (Hann et al., 1999). The final sample was 198 children (111 girls and 87 boys) between 9 and 15 years of age (*M* = 11.7; *SD* = 1.14), enrolled in the 4th grade (*n* = 59; 29.8%), 5th grade (*n* = 101; 51.0%), and 6th grade (*n* = 38; 19.2%). According to the rule of thumb recommended by Kline (1998), an adequate sample size should be 10 times the number of the parameters in path analysis, and the best sample size should be 20 times the number of parameters. To test our proposed mediation model with 11 parameters, a minimum sample size of 110 would be required, and the best sample size would be 220. Hence, the final sample size in this study was sufficient for path analysis.

### Procedure

Permission was acquired from the school principals to conduct the survey in their schools. No parents opted out or indicated disagreement. Eligible migrant children were first explained what the study was about and then were asked whether they would like to participate in the study. Those who agreed to take part in this study were given questionnaires to fill in. The questionnaires were self-administered, including a battery of validated scales measuring stressful experiences, threat and challenge mindsets, coping strategies, and depressive symptoms, as well as questions about basic demographic information. Children took about 30 min to finish the questionnaires. The study protocol was approved by the principal of each migrant school and the Institutional Review Board.

### Measures

All the questionnaires were administered in Chinese.

#### Stressful Experiences

We selected 19 negative life events that were relevant to migrant children, from the 27-item Adolescent Self-Rating Life Events Checklist (ASLEC; Liu et al., 1997), to measure migrant children’s stressful experiences. Stressful life events include interpersonal relationship with peers, teachers and parents (e.g., “*I am not liked by my classmates*,” “*I think my teacher is unfair to me*,” and “*I always have conflicts with my parents*”), academic pressure (e.g., “*I have poor scores in examinations*”), economic stress (e.g., “*I do not have the money to buy what I want*” and “*I always moved house, and my house conditions are poor*”), harsh punishment (e.g., “*I was criticized and punished by my teacher*”), health and adaption (e.g., “*I do not know what to do when I am sick*”), as well as planning and future (e.g., “*I am worried about my future*”). Children reported the degree to which each life event was applicable to their lives, on a five-point Likert-style scale ranging from 1 (very inapplicable) to 5 (very applicable). The average score of all the items was computed to indicate the extent to which migrant children had been exposed to stressful life events, with higher scores indicating more stressful experiences.

#### Stress Mindsets

Threat and challenge mindsets were measured by the Chinese Making Sense of Adversity Scale (CMSAS; Pan et al., 2008). Threat mindset was measured by four items such as “*Adversity means the end of the world and I am not able to resolve it*” and “*Adversity makes me feel that life is meaningless*.” Challenge mindset was measured by eight items such as “*Adversity provides a good opportunity for learning*” and “*Adversity is a fact of life and one cannot grow up without it*.” Migrant children rated the extent to which they agreed with each statement about making sense of adversity on a four-point scale, ranging from 1 (totally disagree) to 4 (totally agree). The average score of the four items in the threat mindset subscale was calculated to indicate threat mindset, and the mean of the eight items in the challenge mindset subscale was analyzed as an indicator of challenge mindset, with higher scores suggesting greater threat mindset and challenge mindset, respectively. Both the threat mindset subscale (Cronbach’s *α* = 0.70) and the challenge mindset subscale (Cronbach’s *α* = 0.71) exhibited acceptable internal consistencies.

#### Coping Styles

Coping styles were assessed by the Brief-COPE (Carver, 1997), which has been widely used to capture one’s effective and ineffective ways to cope with stressful life events. Migrant children reported how often they used each of the 28 coping strategies, on a four-point scale ranging from 1 (I have not been doing this at all) to 4 (I have been doing this a lot). Eisenberg et al. (2012) classified coping strategies measured by the Brief-COPE as (a) approach coping, characterized by the subscales of active coping, positive reframing, planning, acceptance, seeking emotional support, and seeking informational support, and (b) avoidant coping, characterized by the subscales of denial, substance use, venting, behavioral disengagement, self-distraction, and self-blame. The items of humor and religion were excluded because they did not fall into either of the two broad coping styles (Eisenberg et al., 2012). The average score of all the items under each board coping style was computed to indicate approach coping (Cronbach’s *α* = 0.71) and avoidant coping (Cronbach’s *α* = 0.72).

#### Depression

The 20-item Centre for Epidemiologic Studies Depression (CES-D) scale (Radloff, 1977) was adopted to assess the depression levels of migrant children. CES-D is a well-established self-report scale designed to measure depressive symptomatology. Migrant children reported how they have felt or behaved (e.g., “*I did not feel like eating*” and “*I thought my life had been a failure*”) during the past week on a four-point Likert-style scale, where “0” indicated “rarely or none of the time (less than 1 day),” “1” indicated “some or a little of the time (1–2 days),” “2” indicated “occasionally or a moderate amount of time (3–4 days),” and “3” indicated “most or all of the time (5–7 days).” Scores of all items were summed to form an index on depression. Total score ranged from 0 to 60, with a score of 17 or higher indicative of depression. In particular, scores from 17 to 23 suggest mild depression, scores from 24 to 28 suggest moderate depression, and a score of 29 and higher indicates severe depression. The CES-D scale possessed a good internal reliability in the current sample (Cronbach’s *α* = 0.80).

### Statistical Analysis

Pearson correlations were first calculated to examine the bivariate associations among stressful experiences, stress mindsets, coping styles, and depression. Gender difference and age difference in all the main variables were examined by a series of 2 (gender: male vs. female) X 2 (Age group: pre-teens vs. young teens) analyses of variance (ANOVAs), on IBM SPSS Statistics Version 23. Path analysis was then performed on Mplus version 7.31 (Muthén and Muthén, 1988–2012) to examine the multivariate relationships among the main variables. We used indirect effect commands to examine the mediating roles of threat and challenge mindsets in the association between stressful experiences and depression, and the secondary mediating roles of avoidant and approach coping in the relationship between stress mindsets and depression. Gender (dummy coded as 1 = girl and 0 = boy) and age were controlled in the mediation model. Finally, we examined the moderated mediation models by including gender as a moderator in each of the paths in the mediation model. If the moderating effect of gender is significant on a particular path, model constraint commands would be used to test simple slopes. We used the Tucker-Lewis index (TLI) and the comparative fit index (CFI) values above 0.95, as well as the root mean square error of approximation (RMSEA) and the standardized root mean square residual (SRMR) values below 0.05 to indicate excellent model fit. Chi-square value was presented for completeness’s sake.

## Results

### Descriptive Results and Prevalence of Depression

Characteristics of the study sample and the descriptive statistics are presented in Table 1. Assessed by CES-D, 49% of our sample (*n* = 97; 60 girls; *M_age_* = 11.6; *SD* = 0.12) were at risk of depression (scored 17 and above in the CES-D scale). More specifically, 26.8% exhibited mild depression (scored 17–23; *n* = 53; 31 girls; *M_age_* = 11.7; *SD* = 1.20), 12.1% showed moderate depression (scored 24–28; *n* = 24; 15 girls; *M_age_* = 12.4; *SD* = 0.82), and 10.1% fell into the severe depression range (scored 29 and above; *n* = 20; 14 girls; *M_age_* = 11.8; *SD* = 1.24).

**Table 1:** Characteristics of the study sample and descriptive statistics of the main variables.

	Pre-Teens		Young Teens		Total		
	Boys	Girls	Boys	Girls	Boys	Girls	Total
Total n (%)	40 (20.2)	41 (20.7)	47 (23.7)	68 (34.3)	87 (43.9)	111 (56.1)	198 (100.0)
**Age in years**							
Range	9–11	9–11	12–15	12–15	9–15	9–15	9–15
Mean (SD)	10.6 (0.54)	10.7 (0.58)	12.5 (0.72)	12.5 (0.76)	11.7 (1.13)	11.8 (1.14)	11.8 (1.14)
**Grade**							
4th n (%)	23 (57.5)	21 (51.2)	2 (4.26)	13 (19.1)	25 (28.4)	34 (30.4)	59 (29.5)
5th n (%)	17 (42.5)	20 (48.8)	23 (48.9)	39 (57.4)	41 (46.6)	62 (55.4)	103 (51.5)
6th n (%)	0 (0.00)	0 (0.00)	22 (46.8)	16 (23.5)	22 (25.0)	16 (14.3)	38 (19.0)
**ASLEC**							
Negative life events	2.80 (0.64)	3.00 (0.73)	2.71 (0.78)	2.96 (0.86)^a^	2.75 (0.72)	2.96 (0.76)^a^	2.87 (0.75)
**CMSAS**							
Challenge Mindset	2.81 (0.52)	2.70 (0.54)	2.95 (0.51)	2.72 (0.46)^b^	3.02 (0.08)	2.70 (0.51)^b^	2.78 (0.52)
Threat Mindset	2.15 (0.40)	2.05 (0.63)	1.87 (0.61)	2.14 (0.56)^a^	1.98 (0.54)	2.09 (0.59)	2.04 (0.57)
**Brief-COPE**							
Approach coping	2.85 (0.43)	2.70 (0.42)	2.85 (0.42)	2.74 (0.42)^b^	2.84 (0.43)	2.71 (0.42)^b^	2.77 (0.43)
Avoidant coping	2.34 (0.41)	2.29(0.43)	2.10 (0.36)	2.32 (0.39)^a^	2.20 (0.40)	2.31 (0.40)^a+^	2.26 (0.40)
**CES-D**							
Depression score	15.6 (8.30)	17.2 (9.06)	17.2 (9.06)	19.0 (8.43)^a^	15.8 (8.25)	18.3 (8.60)^a^	17.2 (8.53)
Depressed *n* (%)	14 (35.0)	22 (53.7)	23 (48.9)	37 (54.4)	37 (42.5)	60 (54.1)	97 (49.0)

ASLEC, Adolescent Self-Rating Life Events Checklist; CMSAS, Chinese Making Sense of Adversity Scale; CES-D, Centre for Epidemiologic Studies Depression scale.

a Significantly higher than male counterparts at 0.05 level.

b Significantly lower than male counterparts at 0.05 level.

a+ Marginally significantly higher than male counterparts at 0.10 level.

### Bivariate Correlations

Bivariate correlations are displayed in Table 2. As expected, depression was positively associated with self-reported stressful experiences, threat mindset, and avoidant coping, and it was negatively associated with challenge mindset and approach coping. The frequency of experiencing negative life events was positively correlated with threat mindset and avoidant coping, but it was not correlated with challenge mindset or approach coping. As expected, we observed a positive association between threat mindset and avoidant coping, and a positive association between challenge mindset and approach coping. Age was not correlated with any other variables.

**Table 2:** Correlations among the main variables in migrant children.

	1 Stressful experiences	2 Challenge mindset	3 Threat mindset	4 Approach coping	5 Avoidant coping	6 Depression score	7 Age	8 Gender (Girl)
1	-	0.023	0.14^*^	−0.06	0.15^*^	0.29^***^	−0.03	0.12^+^
2		-	−0.19^**^	0.44^***^	−0.11	−0.22^**^	0.07	−0.18^*^
3			-	−0.12	0.35^***^	0.28^***^	0.01	0.09
4				-	0.012	−0.24^**^	0.05	−0.16^*^
5					-	0.24^**^	−0.006	0.12^+^
6						-	0.10	0.15^*^
7							-	0.08
8								-
*N*	198	198	198	198	198	198	196	198
*M*	2.87	2.78	2.05	2.78	2.26	17.2	0.88	56.1%
*SD*	0.77	0.52	0.57	0.43	0.40	8.52	0.44	

*** *p* < 0.001;

** *p* < 0.01;

* *p* < 0.05;

+ *p* < 0.10.

### Gender and Age Differences

A series of 2 (gender: female vs. male) × 2 (Age: pre-teens vs. young teens) ANOVAs was conducted to examine the effects of gender and age on all main variables. Participants were split into two age groups, namely, pre-teens (*N* = 81; 50.6% girls; *M_age_* = 10.6; *SD* = 0.56; range 9–11 years) and young teens (*N* = 115; 59.1% girls; *M_age_* = 12.5; *SD* = 0.74; aged 12–15 years). Two cases were excluded from ANOVAs due to missing age information.

No significant main effect of age group was found on any of the main variables (*p* > 0.10). The main effect of gender was significant on stressful experiences [*F*(1, 195) = 4.00, *p* = 0.047, *η*^2^ = 0.02], challenge mindset [*F*(1, 195) = 5.70, *p* = 0.02, *η*^2^ = 0.03], approach coping [*F*(1, 195) = 4.62, *p* = 0.03, *η*^2^ = 0.02], and depression [*F*(1, 195) = 4.44, *p* = 0.036, *η*^2^ = 0.022], but it was not significant on avoidant coping [*F*(1, 195) = 3.12, *p* = 0.079, *η*^2^ = 0.02] or threat mindset [*F*(1, 195) = 1.08, *p* = 0.30, *η*^2^ = 0.006]. The interaction effects between age group and gender were only significant on threat mindset [*F*(1, 194) = 5.46, *p* = 0.02, *η*^2^ = 0.03] and avoidant coping [*F*(1, 195) = 4.96, *p* = 0.03, *η*^2^ = 0.03], but not on others (*p* > 0.10). In the whole sample, girls reported more stressful experiences, lower levels of challenge mindset, fewer approach coping strategies, and higher levels of depression compared with boys. Among young teens, girls displayed significantly higher levels of threat mindset [*F*(1, 114) = 5.84, *p* = 0.02, *η*^2^ = 0.05] and avoidant coping [*F*(1, 114) = 9.97, *p* = 0.002, *η*^2^ = 0.08] than boys, while the gender differences in these two variables did not occur in pre-teens.

### Mediating Roles of Stress Mindset and Coping Styles in the Association Between Stressful Experiences and Depression

**Figure 1:**
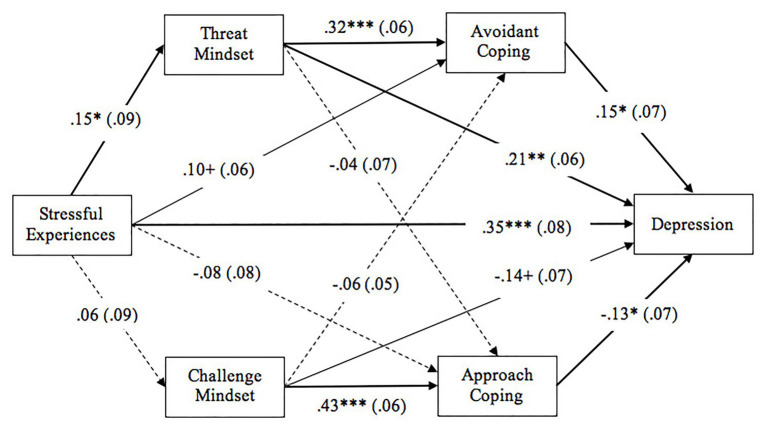
The dual-pathway model of stress coping. Standard errors (SEs) are displayed in the parentheses. Bold lines indicate statistically significant relationship at *p* < 0.05. Normal lines indicate marginal relationship at *p* < 0.10. Dashed lines indicate nonsignificant paths. ^***^p < 0.001; ^**^p < 0.01; ^*^p < 0.05; ^+^*p* < 0.10.

Path analysis was conducted to explore the multivariate relationships among stressful experiences, threat and challenge mindsets, approach and avoidant coping styles, and depression. Gender (dummy coded as 1 = girl and 0 = boy) and age were controlled in this model. As displayed in Figure 1, a dual-pathway mediation model was established with a good model fit, *χ*^2^(1) = 0.49, *p* = 0.48, CFI = 0.99, TLI = 1.05, SRMR = 0.009, RMSEA < 0.001 [90% *CI* = (0, 0.17)]. Stressful experiences showed a direct effect on depression, and this association was mediated by threat mindset [indirect effect: *β* = 0.02, *SE* = 0.01, 95% *CI* (0.0005, 0.05)], but not by challenge mindset (*p* > 0.10). Furthermore, the association between threat mindset and depression was mediated by avoidant coping [indirect effect: *β* = 0.004, *SE* = 0.003, 95% *CI* (0.0001, 0.01)], but not by approach coping (*p* > 0.10). We named this pathway as a *stress-threat-avoidance-depression* pathway, in which threat mindset and avoidant coping mediated the relation of stressful experiences to intensified depression. Somewhat independent from the first pathway, challenge mindset was marginally associated with lower depression scores, and this relationship was mediated by approach coping [indirect effect: *β* = −0.07, *SE* = 0.03, 95% *CI* (−0.15, −0.03)], but not by avoidant coping (*p* > 0.10). We named the second pathway as a *challenge-approach-enhancement* pathway, in which challenge mindset and approach coping contributed to reduced depression, without being associated with stressful experiences. In short, the two distinct stress coping pathways, one stress-threat-avoidance-depression pathway and one challenge-approach-enhancement pathway, may account for the different depression levels of migrant children. A total of 23.8% of the variance in depression levels was explained by these variables.

### Nonsignificant Moderating Role of Gender in the Mediation Model

We further investigated whether gender moderated any mediating path in the dual-pathway stress coping model. Given that the two pathways were somewhat independent, two moderated mediation models were examined. Gender was entered as a moderator in the stress-threat-avoidance-depression pathway, and the challenge-approach-enhancement pathway, respectively. However, gender did not moderate any paths in the two pathways (interaction effects: *p* > 0.10). Neither of the two moderated mediation models obtained a good model fit [model 1: *χ*^2^(3) = 443.1, *p* < 0.001, CFI = 0.37, TLI = −3.39, SRMR = 0.063, RMSEA = 0.87, 90% *CI* (0.80, 0.94); Model 2: *χ*^2^(1) = 95.2, *p* < 0.001, CFI = 0.38, TLI = −5.80, SRMR = 0.012, RMSEA = 0.70, 90% *CI* (0.58, 0.82)]. Results indicated that the two stress coping pathways to depression did not vary by gender.

## Discussion

The current investigation has extended the stress coping research on migrant children by directly addressing how stress mindsets and coping styles work with each other to account for the influence of stressful experiences on depression. Two distinct pathways explained how migrant children achieved differential psychological outcomes. Stress-threat-avoidance-depression pathway is a reactive mechanism, during which experiencing more stressful life events was related to intensified depression levels through threat mindset (that focuses on the damage and loss) and avoidant coping strategies. The challenge-approach-enhancement pathway is a proactive mechanism that might counteract the negative impacts of stressful experiences: Challenge mindset (that focuses on the opportunities of personal growth) was related to fewer depressive symptoms by promoting approach coping strategies, without being associated with stressful experiences. The dual-pathway mechanism did not vary by gender, and it may explain the greater vulnerability of girls to depression.

Migrant children in our sample exhibited a high risk for depression. Forty-nine percent of them were at risk of depression. In particular, 26.8% of the whole sample had mild depression, 12.1% displayed moderate depression, and 10.1% suffered from severe depression. The prevalence of depression in our sample was much higher than that in the average Chinese children and adolescents in China, which was about 20% (Zhou et al., 2018; Rao et al., 2019). This finding was somewhat consistent with previous findings that migrant children displayed more mental health issues such as depression than ordinary children (Hu et al., 2014; Zhou et al., 2018). In line with the prior studies (e.g., Jiang et al., 2019), stressful experiences had a strong direct effect on depression, suggesting that the higher risk of depression among migrant children may be explained by their larger exposure to negative life events. In addition, our sample from private migrant schools appeared to have a higher risk for depression compared with previous reports on a mixed sample of migrant children from both public schools and private migrant schools in Beijing (Ye et al., 2016). In fact, children enrolled in private migrant schools experienced more negative life events and adaption challenges than migrant children enrolled in local public schools (Wang, 2008; Chen et al., 2009; Chen and Feng, 2013; Gao et al., 2015). The greater exposure to adversities might explain why migrant children in our study showed more depressive symptoms than the average migrant children in Beijing (Gao et al., 2015; Jiang et al., 2019).

More importantly, the dual-pathway model of stress coping can help illustrate the mechanism underlying the influences of stressful experiences on depression among migrant children. The two distinct pathways have not only supported but also extended the existing stress coping theories, by revealing how stress mindsets (threat vs. challenge) work with coping styles (avoidant vs. approach) to mediate or mitigate the association between stressful experiences and depression.

The stress-threat-avoidance-depression pathway has supported the transactional theory of stress coping (Lazarus and Folkman, 1984, 1987), which posited stress appraisal and coping as two mediating processes in the relationship between stressful experiences and emotion. Individuals who consistently encounter stressful events tended to anticipate threat and failure from overcoming stress (Rapee and Heimberg, 1997), and people with threat appraisal tended to deploy avoidant coping strategies to deal with stress, which further predicted adjustment problems (Lengua et al., 1999; Lengua and Long, 2002; Zalewski et al., 2011). In children and adolescents, depression was also contributed to by threat appraisal (Bryant et al., 2007; Ellis et al., 2009; Meiser-Stedman et al., 2009) and avoidant coping strategies (Herman-Stabl et al., 1995; Seiffge-Krenke and Klessinger, 2000). We replicated this well-established link in a special young population, rural-to-urban migrant children. Being exposed to more stressful events, migrant children were more likely to interpret stressful situations as more threatening, and adopt more avoidant coping strategies (such as self-blaming, substance use and behavioral disengagement) to deal with stress and, consequently, showed more depressive symptoms. Our findings aligned with previous findings that depressive symptoms of Chinese migrant children were explained by their stress-is-debilitating mindset (Jiang et al., 2019) and maladaptive coping (Ye et al., 2016). The present study linked these paths up to establish a avoidance-motivated mechanism. This stress-threat-avoidance-depression pathway suggests that some risk factors for depression (i.e., exposure to negative life events, threat mindset, and avoidant coping strategies) should be taken into account when designing intervention programs for this vulnerable young population.

The challenge-approach-enhancement pathway has supported the theories of resilience, which emphasized that individuals can utilize personal attributes and personal competencies to counteract the negative influence of adversities on psychological well-being (Garmezy et al., 1984; Masten et al., 1990). The prior studies on Chinese migrant children demonstrated that experiencing more stressful life events does not necessarily aggravate depressive symptoms, especially among children with resilience and positive stress mindset (Ye et al., 2016; Jiang et al., 2019). Our study added that challenge mindset and approach coping strategies can act as protective factors to mitigate the impacts of stressful experiences on depression. Without being influenced by stressful experiences, migrant children with a challenge mindset tended to focus on the potential benefits of stress and use more approach coping strategies (such as positive reframing, planning, and seeking emotional and informational support) to deal with stress, which then alleviated their depressive symptoms. Our results were in line with previous findings that one’s positive psychological outcomes can be explained by challenge appraisal (Blascovich and Tomaka, 1996; Blascovich et al., 2000) and approach coping strategies (Herman-Stabl et al., 1995; Lengua et al., 1999). Additionally, the association between challenge appraisal and approach coping among children and adolescents (e.g., Lengua and Long, 2002; Zalewski et al., 2011) has been extended to migrant children in this study. The challenge-approach-enhancement pathway is crucial because it emphasized personal resilience to positively adapt to a variety of adversities, which are usually inevitable for rural-to-urban children (e.g., discrimination from local residents and disparity of resources). Taken together, enhancing migrant children’s challenge mindset to acknowledge the opportunities of personal growth inherent in stress, and promoting their approach coping strategies, can be integrated to resilience-based intervention programs.

The distinct roles of threat and challenge mindsets in the dual-pathway model of stress coping may be explained by the information process model of anxiety of Beck and Clark (1997). In the initial stage of processing a stimulus, threat impression and automatic negative thoughts occur rapidly as a reactive process, whereas challenge appraisal usually occurs in a later stage where individuals engage in a more reflective process. Hence, after frequently processing stressful stimuli, migrant children may have repeatedly activated automatic negative thoughts and threat appraisal, and build up a meta-cognitive belief that stress has a threatening and debilitating nature, in general, which might lead them to avoidance-motivated responses. This avoidance-motivated process may be activated automatically in responses to stressful situations. However, during the secondary elaboration stage, when actively reappraising the situations and reflecting on personal resources, some migrant children may modify their interpretation bias and find the potential benefits and gains from overcoming stressful events, and eventually develop a positive belief that stress has an enhancing nature in general, which may then lead them to approach-motivated responses. Indeed, previous research has shown that, although experiencing negative life events frequently, some migrant children still hold a positive belief that stress has enhancing consequences (Jiang et al., 2019), and they were able to bounce back with positive psychological well-being (Ye et al., 2016). To sum up, despite the exposure to negative life events, if migrant children can develop a positive stress mindset and use approach coping strategies to deal with stress, they can still achieve positive emotional and adjustment outcomes.

Notably, the dual pathways to depression did not vary by gender, and instead, they explained why girls were more vulnerable to depression. Consistent with some prior studies on rural-to-migrant children (Hu et al., 2014; Jiang et al., 2019), girls in our sample showed a higher risk for depression than boys. We took a further step to suggest that migrant girls’ vulnerability to depression might be explained by greater environmental adversity, negative stress mindset (i.e., lower challenge mindset and higher threat mindset), and maladaptive coping style (i.e., fewer approach coping strategies and more avoidant coping strategies). The findings were supportive of research in other samples (for reviews, see Nolen-Hoeksema et al., 1999; Nolen-Hoeksema, 2001) and the Cognitive Vulnerability-Transactional Stress Depression Model (Hankin and Abramson, 2001). Moreover, from the developmental perspective, we found that gender differences in depression, stressful experiences, challenge mindset, and approach coping can occur as early as in pre-adolescence (e.g., 9–11 years), while gender differences in threat mindset and avoidant strategies only emerged in early adolescence (e.g., 12–15 years). Practically speaking, although all migrant children need support to enhance psychological well-being, girls may require additional attention, especially to their environment, interpretation styles, and coping resources.

The present research has made some contributions to the field. Theoretically, this study has extended the existing theories of stress coping and resilience by revealing that threat mindset works with avoidant coping to mediate, while challenge mindset works with approach coping to mitigate, the impacts of stressful experiences on depression. It has advanced our understanding of the mechanism by which negative life events influence migrant children’s depression levels. Practically, the current investigation highlighted some potential risk factors (e.g., exposure to negative life events, threat mindset, and avoidant coping) and protective factors (e.g., challenge mindset and approach coping) for depression. Findings have provided insights into resilience-based intervention programs, which focus on boosting protective factors such as individual attributes, individual competencies, as well as environmental resources to promote psychological well-being among children and adolescents (Dray et al., 2017), and migrant children in China (Tam et al., 2020). For example, eliminating the risk factors (e.g., increasing supports from family, school, community, and the society to reduce adversities, modifying threat mindset, and terminating avoidant coping strategies) as well as promoting child-level protective factors, such as challenge mindset and approach coping strategies, may facilitate migrant children to achieve positive psychological well-being.

There are several limitations that may guide future research. First of all, all the participants were recruited by convenience sampling from two private migrant schools in Beijing. Without a comparison between migrant children in private migrant schools and those enrolled in local public schools, we cannot generalize our findings to migrant children in public schools, which was proposed as a protective environment (Gao et al., 2015; Jiang et al., 2019). Relatedly, the comparison between rural-to-urban migrant children and non-migrant children in urban areas was lacking, so whether the dual-pathway stress coping model applies to typically developing children is unknown. Second, we did not collect information on family socioeconomic status (SES) or migration details such as the length of time in Beijing, living arrangement with one or two parents, and previous left-behind experiences in rural areas, which should be controlled in our stress coping model. Moreover, the insufficient statistical power resulted from a relatively small sample size required us to be more cautious when interpreting the results. Additionally, all the variables were collected by self-reported measures, so we need to take response bias like social desirability into account. Last but not the least, the cross-sectional results cannot offer strong support for a directional interpretation of causal relationship. Therefore, future studies will benefit from (1) comparing migrant children from private migrant schools with migrant children from local public schools, left-behind children in rural areas, and typically developing children in urban area, to examine whether the dual-pathway stress coping model also applies to other samples, (2) controlling for family SES (e.g., parents’ education and family income), migration-related variables (e.g., length of migration, left-behind experiences in rural areas, and living arrangement in destination), and environmental factors (e.g., school types) in the stress coping model, (3) enlarging sample size to get sufficient statistical power, (4) including implicit measures, biomarkers, observations, or informant ratings to assess stress mindset, emotion and coping strategies, as well as (5) deploying an experimental design to examine the causal relationships among the main variables, or using longitudinal data to explore the long-term effects of stress mindsets and coping on migrant children’s mental health.

## Conclusion

The current investigation discovered a dual-pathway stress coping model, with one stress-threat-avoidance-depression pathway and one challenge-approach-enhancement pathway, to explain the different depression levels among rural-to-urban migrant children in China. As a reactive process, threat mindset and avoidant coping mediated the impacts of stressful experiences on aggravated depressive symptoms. In a proactive process, without being influenced by stressful experiences, challenge mindset promoted approach coping, which then alleviated depressive symptoms. The two stress coping pathways did not vary by gender, and instead, they explained the greater vulnerability of girls to depression. Finally, the risk factors (e.g., stressful experiences, threat mindset, and avoidant coping) and protective factors (e.g., challenge mindset and approach coping) for depression have shed light on future resilience-based interventions to mitigate the negative impacts of negative life events and promote migrant children’s psychological well-being.

## Data Availability Statement

The raw data supporting the conclusions of this article will be made available by the authors, without undue reservation.

## Ethics Statement

The studies involving human participants were reviewed and approved by Beijing Normal University, China. Written informed consent to participate in this study was provided by the participants’ legal guardian/next of kin.

## Author Contributions

LC: conception of the work, study design, data collection, data analysis, and draft of the manuscript. LC and LQ: interpretation of data and revision of the manuscript. All authors contributed to the article and approved the submitted version.

### Conflict of Interest

The authors declare that the research was conducted in the absence of any commercial or financial relationships that could be construed as a potential conflict of interest.
